# Injectable PLGA/Fe_3_O_4_ implants carrying cisplatin for synergistic magnetic hyperthermal ablation of rabbit VX2 tumor

**DOI:** 10.1371/journal.pone.0177049

**Published:** 2017-05-04

**Authors:** Yang Yang, Fengjuan Wang, Kaiyuan Zheng, Liming Deng, Lu Yang, Nan Zhang, Chunyan Xu, Haitao Ran, Zhaoxia Wang, Zhigang Wang, Yuanyi Zheng

**Affiliations:** 1 Chongqing Key Laboratory of Ultrasound Molecular Imaging, Second Affiliated Hospital of Chongqing Medical University, Chongqing, China; 2 Department of Nephrology, Chongqing People’s Hospital, Chongqing, China; 3 Department of Ultrasound, Children’s Hospital of Chongqing Medical University, Chongqing, China; 4 Shanghai Institute of Ultrasound in Medicine, Shanghai Jiao Tong University affiliated Shanghai Sixth People's Hospital, Shanghai, China; Shanghai Tenth People's Hospital, CHINA

## Abstract

Magnetic hyperthermia ablation has attracted wide attention in tumor therapy for its minimal invasion. Although the chemo-hyperthermal synergism has been proven to be effective in subcutaneously xenografted tumors of nude mice in our previous experiment, the occurrence of residual tumors due to incomplete ablation is more common in relatively larger and deeper-seated tumors in anti-tumor therapy. Thus, a larger tumor and larger animal model are needed for further study of the therapeutic efficacy. In this study, we tested the efficiency of this newly developed technique using a rabbit tumor model. Furthermore, we chose cisplatin (DDP), which has been confirmed with high efficiency in enhancing hyperthermia therapy as the chemotherapeutic drug for the synergistic magnetic hyperthermal ablation therapy of tumors. In vitro studies demonstrated that developed DDP-loaded magnetic implants (DDP/PLGA-Fe_3_O_4_) have great heating efficacy and the drug release can be significantly boosted by an external alternating magnetic field (AMF). In vivo studies showed that the phase-transitional DDP/PLGA-Fe_3_O_4_ materials that are ultrasound (US) and computerized tomography (CT) visible can be well confined in the tumor tissues after injection. When exposed to AMF, efficient hyperthermia was induced, which led to the cancer cells’ coagulative necrosis and accelerating release of the drug to kill residual tumors. Furthermore, an activated anti-tumor immune system can promote apoptosis of tumor cells. In conclusion, the DDP/PLGA-Fe_3_O_4_ implants can be used efficiently for the combined chemotherapy and magnetic-hyperthermia ablation of rabbit tumors.

## Introduction

Magnetic hyperthermia has been rapidly developed and has attracted ever-increasing attention in cancer treatment because of its unique property of converting electromagnetic energy into heat through the application of magnetic materials subjected to an external AMF to induce cancer cell death noninvasively only for a targeted area with minimal damage to normal tissue[1–4]. The basis of hyperthermia for cancer treatment is the high sensitivity of cancer cells to temperatures ranging from 41°C to 45°C, which can cause tumor cell death, in contrast to normal cells[5]. It is known that the therapeutic effect of hyperthermia depends on the temperature of the targeted region. Thermal ablation with temperatures that exceed 46°C (up to 56°C) has the ability to quickly remove a small tumor through necrosis, coagulation, and carbonization[6, 7]. AMF’s ability to generate heat was determined by the magnetic properties of magnetic materials subjected to AMF. Currently, there are a number of widely used magnetic media for this option, such as magnetic fluids, intravenous magnetic nanoparticles, and ferromagnetic thermoseeds. However, these media have some problems[8, 9] hindering their broad application, including the invasive operation to remove the magnetic thermoseeds due to a lack of degradation in the body, difficulty of accumulation in the target site, easy leakage into the surrounding tissue or blood vessels due to the small particulate sizes, and the need for a large number of magnetic nanoparticles to be implanted invasively because of the poor thermal-transfer efficiency. Thus, obtaining an ideal and efficient magnetic material has remained a challenge in hyperthermal therapy.

In our previous study, we developed an injectable phase transformation in situ forming implants (ISFIs) based on poly (lactic-co-glycolic acid) (PLGA) integrating with Fe powder for the magnetic hyperthermal ablation of tumors[10]. In brief, a biodegradable polymer PLGA is dissolved in a biocompatible organic solvent N-methyl pyrrolidone (NMP), which is initially comprised of a liquid gel solution that undergoes a process of solidification after placement in the aqueous environment. The water-insoluble PLGA precipitates as the water miscible NMP leaches out into the bath solution and water diffuses into the implant, which is referred to as a phase inversion[11, 12]. After injection into tumors, the implant can be restricted in the tumor site as a solid state without leaking to the adjacent normal tissues for a safe treatment. However, like other single-therapy strategies, magnetic hyperthermia for tumors using a PLGA/Fe implant system alone was not sufficient following the risk of residual tumors leading to tumor recurrence. Chemotherapy is one alternative option for tumor treatment. However, the main drawback of conventional chemotherapy is its nonspecific distribution resulting in systemic side effects and reduced therapeutic effects. It is reported that hyperthermia can enhance the therapeutic efficacy of chemotherapy. Therefore, our group developed a multi-therapeutic approach based on PLGA-Fe loading with doxorubicin (DOX) and we have proven the efficiency of the chemo-hyperthermal synergistic therapy for tumors[13].

However, as a pilot experiment, we chose a subcutaneously xenografted tumor with cancer cells (SMMC-7721 human HCC) in nude mice. As is known, the smaller and more superficial the tumor is, the more effective treatment is. Although the chemo-hyperthermal synergism has been verified, for relatively large and deep-seated tumors, residual tumors are more likely to occur due to the difficulty of achieving homogeneous heat dispersion and the inefficacy of drug cytotoxicity. Such a situation is more common in clinical anti-tumor therapy. Thus, for the near future, clinical translation, building an ideal animal tumor model that has similar histological and biological characteristics with human malignant tumors, has important value in further evaluation of the synergistic therapeutic efficacy for tumors[14]. Rabbits are the largest animal species to have the self-tumor strain and are extensively used as a large animal model to study different aspects of tumor behavior including therapy efficacy[15–17]. The VX2 carcinoma is a squamous cell carcinoma that is derived from virus-induced skin papilloma of rabbits and can be implanted in many tissues in the rabbit to establish a tumor model that is similar to the human orthotopic tumor model[18, 19]. Compared to subcutaneously or orthotopically xenografted tumors in nude mice, VX2 carcinoma has many characteristics similar to those of human tumor cells, such as its vascularization[20]. Therefore, the rabbit VX2 tumor model employed in this experiment presents a good comparability in the study of therapy for human malignant tumors. Furthermore, although the anti-cancer treatment via hyperthermia combined with chemotherapeutic drugs was shown to have synergistic effect[21–23], it does not mean every chemotherapeutic drug cytotoxicity can be enhanced when combined with hyperthermia, as reported in many studies[24–26]. The selection of an optimum chemotherapeutic drug to strengthen the synergism is a critical strategy to kill the residual tumor cells in large tumors.

DDP is used as first-line active agent against a number of cancers. It has been shown that DDP is temperature-dependent. In vitro and in vivo studies show that its cytotoxicity increases almost linearly with increasing temperature[27, 28]. Maximum synergism can be achieved without increasing systemic side effects, particularly on the bone marrow and kidneys, with agents not presenting any change in cytotoxicity with higher temperatures such as 5-fluorouracil, amsacrine, and vinca alkaloids. Meanwhile, hyperthermia can overcome acquired drug resistance[29, 30], which means the chemo-sensitivity of the tumor cells can be increased and the resistance to DDP in tumor cells can be suppressed under high temperatures. Itoh Y et al. found that hyperthermia alone (41°C) and a low drug concentration (20 μg/ml of DDP) did not have any cell-killing effect on a human bladder cancer cell line. However, the anti-tumor effect of combination therapy was significantly higher than either hyperthermia or drugs alone. Indeed, the survival rate in the case of the combined treatment was the same as that of a 10-fold higher concentration of DDP administered alone[31]. All of these findings suggest that DDP is one of the best choices when thermal is combined with anti-cancer drugs. As with ISFIs as a drug delivery system[32, 33], DDP may be dissolved in the polymer solution, which can serve as a carrier of both heat and drugs, and be deposited selectively to attain a steady and homogeneous heat distribution within the tumor site when exposed to AMF. Furthermore, studies have indicated that when the temperatures increased to near the glass transition temperature (Tg) of the polymer, the drug release rate would increase three times[34]. Therefore, effective drug release can be achieved simultaneously.

It is known that hyperthermia faces limitations of restricting the heating to the tumor sites after a non-lethal thermal stress and the reduced efficacy of heating, because tumor cells typically acquire resistance to induced thermal stress. This phenomenon is called thermoresistance[35, 36], which has been demonstrated to be adverse to hyperthermia. It has been verified that DDP can reduce thermoresistance and increase the thermosensitivity of tumor cells[37–39].

In this study, we designed injectable, phase-transitional, and ultrasound and CT imaging visible DDP/PLGA-Fe_3_O_4_ materials to maximize the synergistic efficacy in a rabbit large tumor model. When AMF was applied externally, the hyperthermia at the vicinity of the implant induced cancer cell coagulative necrosis and increased the cytotoxicity of the drug and accelerated the drug release to kill residual tumors. This combined treatment modality could provide a promising strategy for improving the therapeutic efficacy in tumors and the use of larger rabbit tumor models further increased the possibility of bench-to-bedside translation.

## Materials and methods

### Preparation of DDP/PLGA-Fe_3_O_4_

DDP (Sigma-Aldrich) was dissolved into NMP (Sigma-Aldrich), and then PLGA (with a 50:50 lactide/glycolide (L/G) ratio, MW38000 Da, Jinan Daigang Biomaterial Co., Ltd) was added into the mixture overnight at 37°C in an incubator-shaker at 100 rpm after a homogenous solution was obtained. The ratio of PLGA and NMP in the DDP/PLGA was 1.1 g: 2 ml. The Fe_3_O_4_ magnetic nanoparticles (Chengdu Ai Keda Chemical Reagent Co., Ltd.) with strong magnetic performance were uniformly dispersed into the DDP/PLGA gel using a simple mechanical stirring. To examine the heating efficiency of the mixture, 3 formulations were prepared containing Fe_3_O_4_ with a mass fraction of 10%, 20%, and 30%, the weight ration of DDP/PLGA and Fe_3_O_4_ was 9:1, 8:2, 7:3, respectively. The ultimate mass fraction of DDP in the gel was 1%.

### Characterization of DDP/PLGA-Fe_3_O_4_

The morphology, microstructure, and composition of DDP/PLGA-Fe_3_O_4_ were investigated via scanning electron microscopy (SEM, AMETEK, S-3700, 371027–02, EDAX, Inc., NJ, USA). Elemental analysis was performed using an energy dispersive X-ray spectrometer (EDS, AMETEK, S-3700, 371027–02, EDAX, Inc., NJ, USA). Solid DDP/PLGA-Fe_3_O_4_ was used as the analyzed sample. The phase-transformation process of DDP/PLGA-Fe_3_O_4_ was recorded with MyLab 90 (Esaote, Italy) using a linear array probe (5–12 MHz) in an agar gel phantom. The gel phantom was made using 2% agar-agar (w/v) dissolved in de-ionized water. An Eppendorf tube was placed in the center of the phantom to create a hole where the DDP/PLGA-Fe_3_O_4_ solution was dropped.

### Evaluation of AMF-induced heating ability in vitro

The DDP/PLGA-Fe_3_O_4_ aqueous solution (50 μl) with a different Fe_3_O_4_ mass fraction (10%, 20%, or 30%) was dropped into the saline solution (1 ml) in the glass tubes. The DDP/PLGA solution containing no Fe_3_O_4_ was used as the blank control. Then the glass tubes were placed at the center of the coil of the homemade magnetic hyperthermia analyzer. The frequency and output current were 626 kHz and 28.6 A, respectively. The temperature of the saline solution was continuously monitored by a far-infrared thermometer (Fluke Ti32, Fluke Corporation, USA). Thermal images were saved every 10 s over a span of 120 s and analyzed via SmartView 3.6 software. The blank control was treated under the same conditions.

### Evaluation of heating efficiency in excised bovine liver

The fresh excised bovine liver was purchased from a slaughterhouse. Based on the above experiments, DDP/PLGA-30% Fe_3_O_4_ was chosen for further study. Liquid DDP/PLGA-30% Fe_3_O_4_ (100 μl) was injected into excised bovine liver cuboids (6 cm × 2 cm × 2 cm) via a syringe under the guidance of ultrasound, then exposed to the same AMF for 3 min. The peak surface temperature of the liver at different distances (0, 1, 2, and 3 cm away from the injection point) was measured at the time point of 180 s by a far-infrared thermometer.

### Evaluation of ablation efficiency in excised bovine liver

Liquid DDP/PLGA-30% Fe_3_O_4_ with different amounts (50 μl or 100 μl, respectively) were injected into the center of the excised bovine liver blocks (2 cm × 2 cm × 2 cm) via syringe under the guidance of ultrasound. After liquid DDP/PLGA-30% Fe_3_O_4_ quickly converted to its solid state, the blocks were exposed to the same AMF for 1 min, 3 min, and 5 min, respectively. The grayscale values before and after ablation were observed by ultrasound images. The temperature of the liver blocks was recorded every 10 s. The ablated tissue volumes were estimated by the following equation:

V=π/6×a×b×c

where *V* is the ablation volume (cm^3^), a is the maximum length of the ablated tissue measured by a ruler, *b* is the maximum width of the ablated tissue measured by a ruler, and *c* is the maximum thickness of the ablated tissue measured by a ruler. Experiments for each group were performed 3 times.

### In vitro AMF-induced drug release from DDP/PLGA-Fe3O4

The drug release profiles of DDP/PLGA-30% Fe_3_O_4_ with and without exposure to AMF were investigated. Prepared liquid DDP/PLGA-30% Fe_3_O_4_ (50 μl) was immersed in 1 ml of phosphate-buffered saline (PBS, PH 7.4) in a dialysis bag (molecular weight cut-off: 8000–14000) and placed in a centrifuge tube (45 ml) with an additional 29 ml of PBS. The centrifuge tubes were completely covered with aluminum foil to avoid exposure to light and then placed in an incubator-shaker at 37°C and 100 rpm to mimic the human tissue or blood. At the time points of 2 and 8 h, the centrifuge tubes were exposed to the AMF for 10 s and 30 s, respectively. One ml of the buffer medium was sampled from each tube at 0.5, 1, 2, 4, 8, 12, 24, 48, 72, and 96 h and 1 ml of fresh PBS was compensated in order to maintain a constant volume of 30 ml. The release solution was derived by diethyldithiocarbamate trihydrate (DDTC) at 37°C and then the organic phase was extracted by trichloromethane. The amount of released DDP was quantified by the high-performance liquid chromatography (HPLC) system (Waters e2695, USA) using an Xbridge C_18_ (3.5 μm, 4.6 mm × 150 mm) column with the mobile phase of acetonitrile and 0.25% sodium chloride solution (71:29, v/v). The detective UV wavelength was 254 nm and the velocity of flow was 0.6 ml/min. The experiments were performed in triplicate to ensure accuracy. The group without AMF treatment was assessed under the same condition as the control. The standard curve has been drawn using the known concentrations of DDP and was used to determine the concentration of DDP released from the DDP/PLGA-Fe_3_O_4_.

### The thermal stability of DDP

The thermal stability of DDP was determined using ultraviolet visible (UV-vis) spectrophotometry. DDP (10 mg) was completely dissolved in 0.9% sodium chloride solution (10 ml) and was then incubated in a water bath at 80°C for 15 min and 30 min, respectively. The solution at room temperature (20°C) was used as a control.

### In vitro biocompatibility and cytotoxicity of DDP/PLGA-Fe_3_O_4_

The human ovarian cancer cell line SKOV3 (Shanghai Institutes for Life Sciences, the Chinese Academy of Sciences) was seeded in a 48-well plate at a density of 5 × 10^4^ cells per well with RPMI 1640 medium in a humidified incubator at 37°C with 5% CO_2_ for 24 h. 50 μl, 100 μl, and 200 μl of PLGA-30% Fe_3_O_4_ (without DDP) were immersed into 10 ml RPMI-1640 medium for 24 h in an incubator at 37°C, respectively, to acquire different concentrations of PLGA-30% Fe_3_O_4_ in RPMI-1640 medium (5 μl/ml, 10 μl/ml, and 20 μl/ml). The RPMI-1640 medium was removed and replaced the culture medium with the different concentrations of immersed solution, followed by incubation for 24 h at 37°C. SKOV3 cancer cells were treated with RPMI-1640 medium as a blank control. The optical density (OD) of each well was measured by a microplate reader (Model 680, Bio-Rad, Inc.) at a wavelength of 490 nm. The cell viability was assessed by the Cell Counting Kit-8 (CCK8) assay.

### Preparation of rabbit VX2 tumor models

All of the animal experimental procedures were carried out in strict accordance with the policy of the Institutional Animal Care and Use Committee (IACUC) of Second Affiliated Hospital of Chongqing Medical University, Chongqing, China, and conformed to the Ministry of Science and Technology of Health Guide for Care and Use of Laboratory Animals (Publication No.398,2006). The protocol was approved by the IACUC of Second Affiliated Hospital of Chongqing Medical University. All surgery in the study was performed under sodium pentobarbital anesthesia, and all efforts were made to minimize suffering. Animals were housing in separate home cages in a clean level environment at room temperature (24°C) with free access to food and water. Viability and behaviour were recorded every day. Body weight was recorded three times weekly. If one of the following conditions occurred during the experiment, the animals were humanely killed considered: signs of suffering (pain, weakening); tumor ulcerating; loss of more than 15% of baseline weight.

Forty-eight New Zealand white rabbits of either gender, 2–2.2 kg weight (Animal experiment center of Chongqing Medical University) were anesthetized by injection with 3% pentobarbital solution via the ear vein (1 ml/kg). The thighs of the rabbits were depilated with 8% Na_2_S solution. The tumor block excised from the New Zealand white rabbits’ VX2 hepatic carcinoma (Animal experiment center of Chongqing Medical University) was sheared into small pieces of approximately 1 mm^3^, then embedded intramuscularly directly in the hind limb using ophthalmological forceps. The wound was sutured and disinfected. To avoid infection, 400,000 units of streptomycin were intramuscularly injected over the 3 days following the operation. The rabbits were used for experiments when the tumor volume grew to approximately 1.40 cm^3^. The dimension of the tumors was measured with US images and the tumor volume was calculated using the following formula:

V=π∕6×a×b×c

where *a*, *b*, and *c* represent the transverse diameter (cm), the vertical diameter (cm), and the anteroposterior diameter (cm) of the tumor tissue, respectively. *V* represents the volume of the tumor (cm^3^).

It has been indicated that the volume of ablated excised bovine liver for 100 μl DDP/PLGA-30% Fe_3_O_4_ under AMF for 3 min was 1.267 ± 0.067 cm^3^. If rabbits bearing VX2 xenograft tumors in the muscle of the hind legs were injected with 100 μl DDP/PLGA-30% Fe_3_O_4_ and exposed to AMF for 3 min, the tumors could not be ablated completely. The residual tumor models were built successfully.

### Study on US/CT visualization in vivo

Rabbits bearing transplanted VX2 tumor were intratumorally injected with 100 μl DDP/PLGA-30% Fe_3_O_4_ via a syringe under the real-time guidance of US (MyLab 90, L5-12 MHz). The images were captured pre-injection, post-injection, and post-ablation (for 3 min) and then imaged under a 16-slice spiral CT (GE Light Speed).

### In vivo magnetic-induced chemo-hyperthermia ablation for tumors

The 48 rabbits with intramural VX2 tumors in their thighs were randomly divided into 4 groups of 12: PLGA/NMP, DDP/PLGA, PLGA-30% Fe_3_O_4_ + AMF, and DDP/PLGA-30% Fe_3_O_4_ + AMF. The rabbits received an intratumoral injection with the same dose of PLGA/NMP, DDP/PLGA, PLGA-30% Fe_3_O_4_, and PLGA/DDP-30% Fe_3_O_4_, respectively. In addition, the PLGA-30% Fe_3_O_4_ and PLGA/DDP-30% Fe_3_O_4_ groups were exposed to AMF for 3 min after administration. Each rabbit was injected and received AMF only once during the experiment. The temperature of tumors was continuously monitored by a far infrared thermometer and recorded every 10 s. Blood samples (1 ml) were collected using the ear artery at days 1, 4 and 7, and were then centrifuged immediately at 3000 rpm for 8 min. The separated serum was collected to detect soluble interleukin-2 receptor (IL-2R) by enzyme-linked immunosorbent assay (ELISA). The size of the tumor was measured by US images. The tumor volume was calculated by the following formula:

V=π∕6×a×b×c

At 1 day after treatment, three rabbits from each group were euthanized randomly by injection with an overdose 3% pentobarbital solution via the ear vein. Tumor pathology examination by hematoxylin and eosin (HE) staining and a transmission electron microscope (TEM) were performed to detect the structural changes of the tumor cells. The expression of heat shock proteins 70 (HSP70) in the tumor tissues at day 1 was detected using immunohistochemical examinations. At 4 days after treatment, three rabbits from each group were euthanized randomly. For the incomplete ablated tumor tissue in each group, the expression of proliferating cell nuclear antigen (PCNA) for tumor cell proliferation assessing was detected by immunohistochemistry. Apoptosis of tumor cell was detected by TdT-mediated dUTP nick end labeling (TUNEL). The proliferating index (PI) and apoptotic index (AI) were calculated. Five equal-sized fields were randomly chosen, and the percentage was calculated as a ratio of the positively stained cell number to the total tumor cell number in each field. At day 7, three rabbits from each group were euthanized randomly. The concentration of platinum in the tumor tissue (center and edge) was detected by inductively coupled plasma atomic emission spectroscopy (ICP-AES) at days 1, 4, and 7. All of the remaining rabbits were fed for further observation of tumor growth trends. At the end of the experiment, animals were humanely killed for ethical reasons on day 21. The mortality of animals during the experiment are shown in S1 Table.

## Results and discussion

### Characterization of DDP/PLGA-Fe_3_O_4_

**Fig 1:**
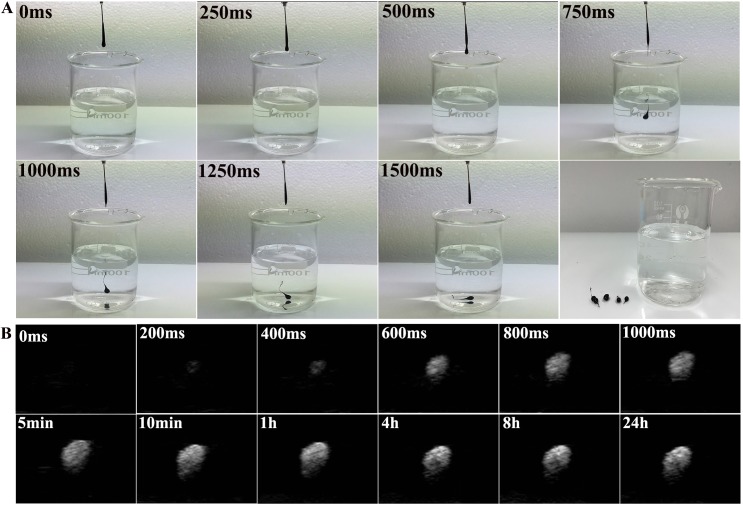
Digital photos and US images of liquid-solid phase transformation of DDP/PLGA-30% Fe_3_O_4_. (A) Digital photos of liquid-solid phase transformation upon contact with water. (B) The echo intensity of DDP/PLGA-30% Fe_3_O_4_ on US images before and after phase transformation at given time intervals in vitro (gel model).

**Fig 2:**
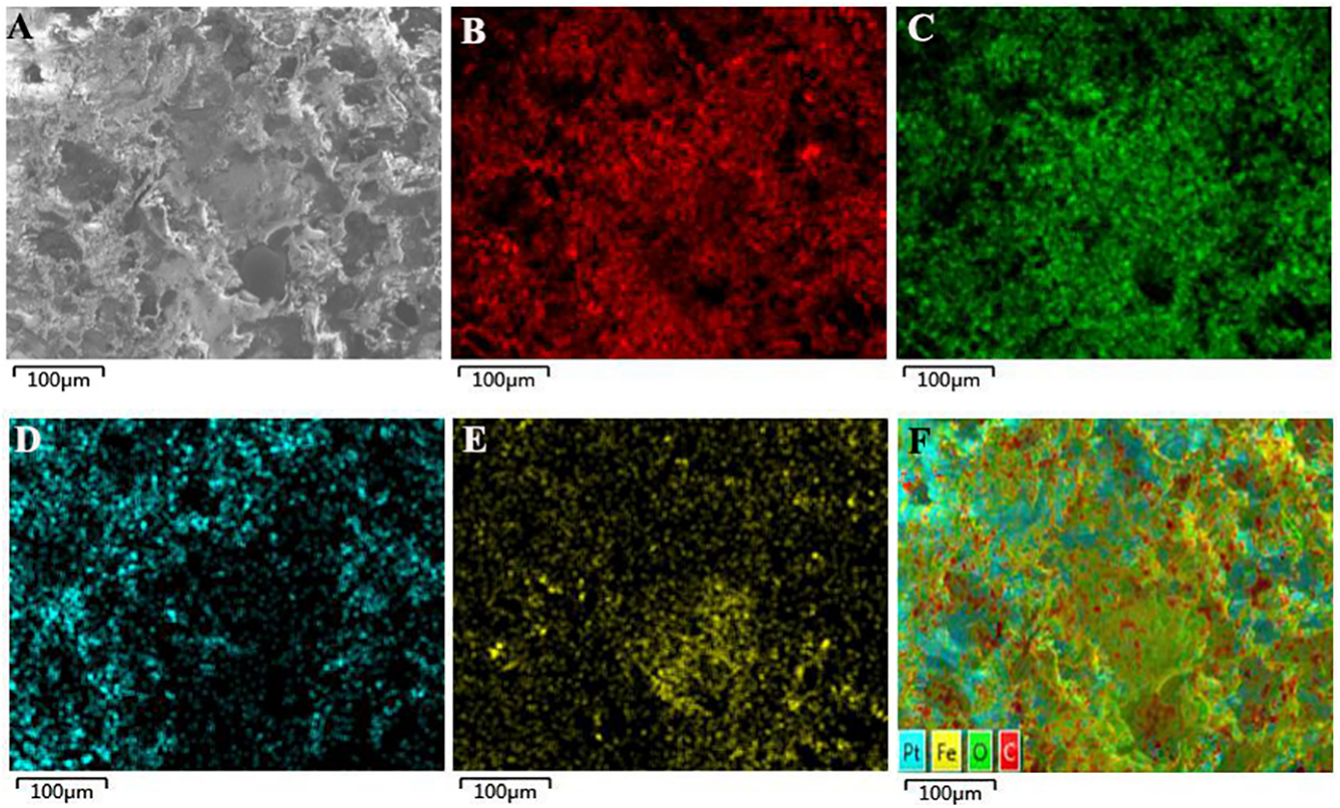
SEM and EDS map scan images for DDP/PLGA-30%Fe_3_O_4_. (A) The SEM image shows that the structure was porous. (B-F) The EDS map scan images show that the Fe element was distributed within the carbonaceous and oxygen, platinum frameworks uniformly. B: C, C: O, D: Pt, E: Fe, F: merged image of B to E.

Liquid DDP/PLGA-Fe_3_O_4_ can be directly injected by a syringe due to its high fluidity. After contact with water, liquid-solid phase transformation occurred immediately (Fig 1A), which ensured the implants would be localized in the tumor tissue. The ultrasound signal intensity of the material was almost unchanged after 1000 ms contact with water, which also demonstrated that the process of the phase transformation was rapid (Fig 1B). Injectability and rapid phase transformation are essential for minimally invasive and safe therapy. The good liquid-solid phase transformation ability can avoid the material penetrating into the surrounding tissues or blood vessels in vivo, and, as a consequence, will prevent the increase of temperature in the normal tissue during the magnetic hyperthermia treatment. SEM images of the solid phase of DDP/PLGA-Fe_3_O_4_ are shown in Fig 2A. The morphology and structure was loose and porous. Fig 2B–2F exhibits that Fe element was well-distributed within the material.

### AMF-induced heating ability in vitro

**Fig 3:**
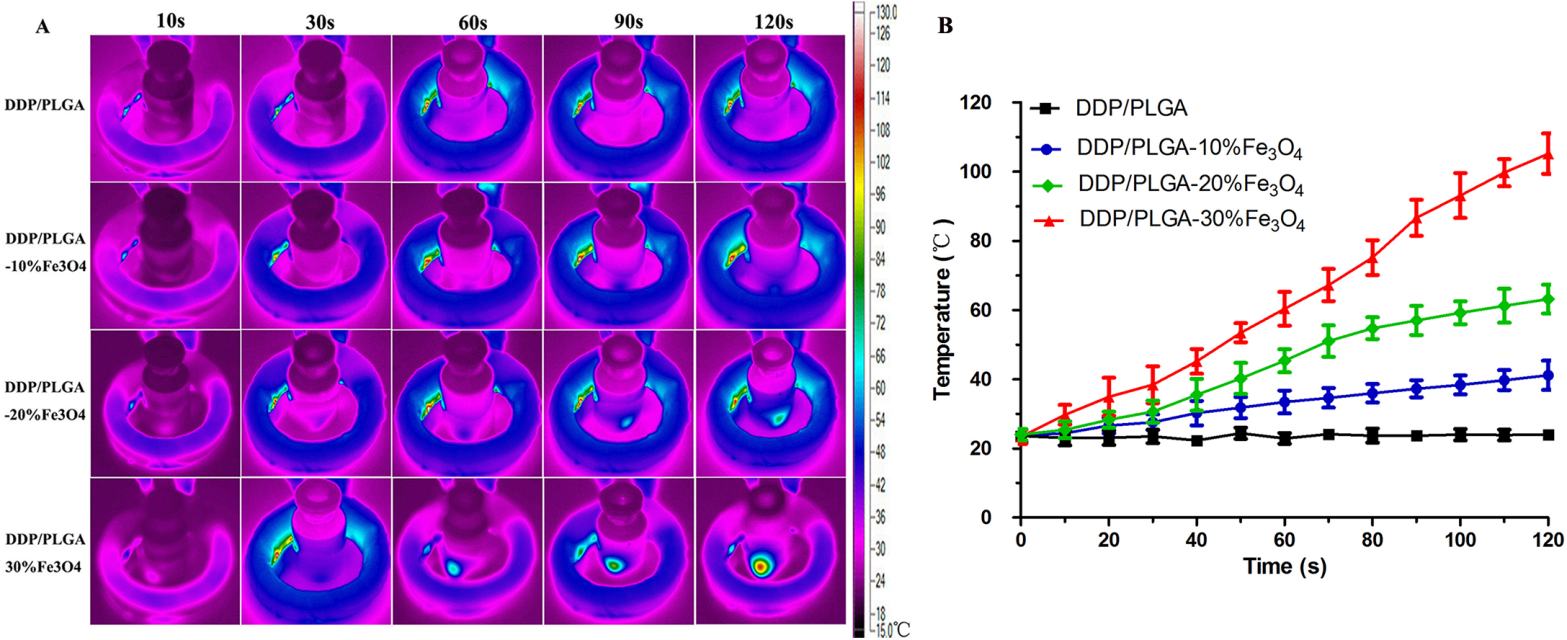
AMF-induced heating ability in PBS solution. (A) Thermal images of DDP/PLGA-Fe_3_O_4_ with a different mass of Fe_3_O_4_ under AMF, PLGA/NMP without Fe_3_O_4_ as a control. (B) The corresponding time-temperature curve of DDP/PLGA-Fe_3_O_4_ with a different mass of Fe_3_O_4_ exposed to AMF for 3 minutes.

The thermal images showed that the temperature of PBS containing DDP/PLGA-Fe_3_O_4_ increased rapidly under AMF while the temperature of the control group without Fe_3_O_4_ was unchanged (Fig 3A), demonstrating that DDP/PLGA-Fe_3_O_4_ has great magnetic thermal conversion efficiency depending on the Fe_3_O_4_ content and extended time of exposure to AMF. The corresponding time-temperature curve of DDP/PLGA-Fe_3_O_4_ containing different Fe_3_O_4_ content under AMF showed that with the increase of Fe_3_O_4_ concentration, the interval time to reach the highest temperature shortened (Fig 3B). When exposed to AMF for 3 minutes, the temperatures in the DDP/PLGA-10% Fe_3_O_4_, DDP/PLGA-20% Fe_3_O_4_, and DDP/PLGA-30% Fe_3_O_4_ groups reached 41.1 ± 4.2°C, 63.2 ± 4.1°C, and 105.2 ± 5.8°C, respectively.

### Heating and ablation efficiency in excised bovine liver

**Fig 4:**
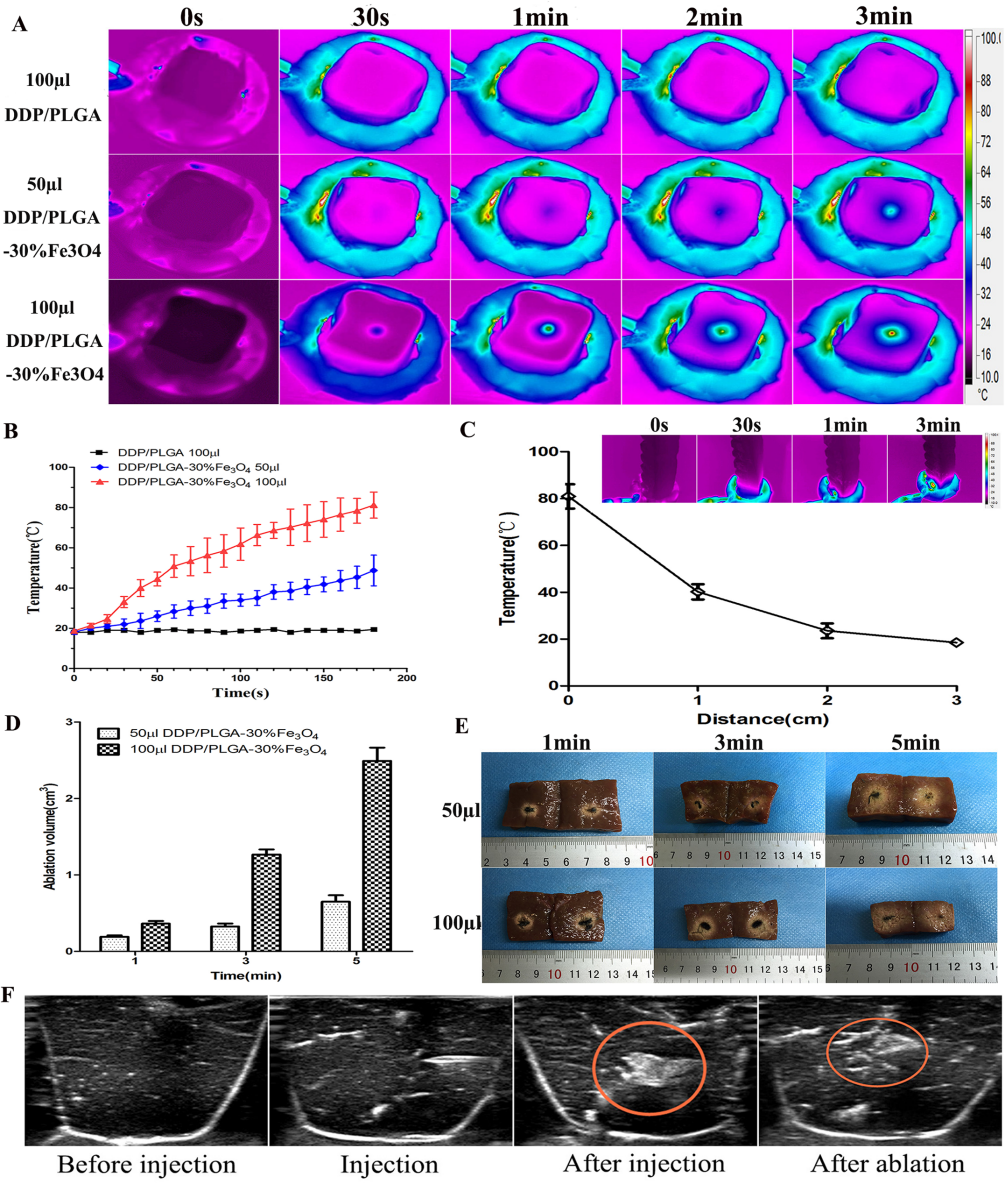
Ablation efficiency of DDP/PLGA-30% Fe_3_O_4_ in excised bovine liver. (A) Thermal images of excised bovine liver containing 50 μl and 100 μl DDP/PLGA-30% Fe_3_O_4_ under AMF for 3 min, with excised bovine liver containing 100 μl DDP/PLGA as a control. (B) The corresponding time-temperature curve of the excised bovine liver exposed to AMF for 3 min. (C) The distance–temperature curve of the excised bovine liver containing 100 μl DDP/PLGA-30% Fe_3_O_4_ after 3 min exposure to AMF. (D) The corresponding ablation volume of the excised bovine liver containing 50 μl and 100 μl DDP/PLGA-30% Fe_3_O_4_ followed by exposure to AMF for 1 min, 3 min, and 5 min, respectively. (E) The corresponding macroscopic photos. (F) US images of excised bovine liver before and after injection with 100 μl DDP/PLGA-30% Fe_3_O_4_ followed by exposure to AMF for 3 min.

Good heating ability is essential for successful thermal ablation. Based on previous experiments that DDP/PLGA-30% Fe_3_O_4_ can quickly generate heat when exposed to AMF, therefore, we evaluated the heating and ablation efficiency in excised bovine liver. As shown in the thermal images (Fig 4A), the surface temperature of the bovine liver increased with prolonged time of exposure to AMF. At the same time point, the surface temperature of bovine liver containing 100 μl DDP/PLGA-30% Fe_3_O_4_ was higher than that containing 50 μl DDP/PLGA-30% Fe_3_O_4_ (Fig 4B). The temperatures for 50 μl DDP/PLGA-30% Fe_3_O_4_ were 28.3 ± 3.3°C, 38.1 ± 3.5°C, and 48.7 ± 5.5°C, at 1 min, 2 min, and 3 min, respectively. The temperatures for 100 μl DDP/PLGA-30% Fe_3_O_4_ were 50.9 ± 5.6°C, 68.6 ± 4.0°C, and 81.2 ± 6.5°C, at 1 min, 2 min, and 3 min, respectively. The thermal images of 6 cm × 2 cm × 2 cm excised bovine liver containing 100 μl DDP/PLGA-30% Fe_3_O_4_ showed that the temperature decreased significantly with the distance to the location of the implants (Fig 4C), decreasing from 80.9 ± 5.6°C (0 cm away from the injection point) to 23.5 ± 3.2°C (2 cm away from the injection point).

The necrotic volumes of ablated bovine livers increased with the mass of the DDP/PLGA-Fe_3_O_4_ and the duration time under AMF (Fig 4D). The ablated volumes for 100 μl DDP/PLGA-30% Fe_3_O_4_ increased from 0.364 ± 0.035 cm^3^ at 1 min to 1.267 ± 0.067 cm^3^ at 3 min under AMF. The ablated volumes for 50 μl DDP/PLGA-30% Fe_3_O_4_ increased from 0.191 ± 0.018 cm^3^ at 1 min to 0.325 ± 0.040 cm^3^ at 3 min under AMF. The ablated liver tissue split into two parts presented a pale color when observed with naked eyes (Fig 4E). Furthermore, as shown in the ultrasound images (Fig 4F), DDP/PLGA-30% Fe_3_O_4_ appeared hyperechoic with posterior shadowing. After ablation, the echo intensity of the tissue around the implants increased. It can be used as an ultrasound contrast agent to guide and monitor the process of therapy.

### In vitro drug release from DDP/PLGA-Fe_3_O_4_

**Fig 5:**
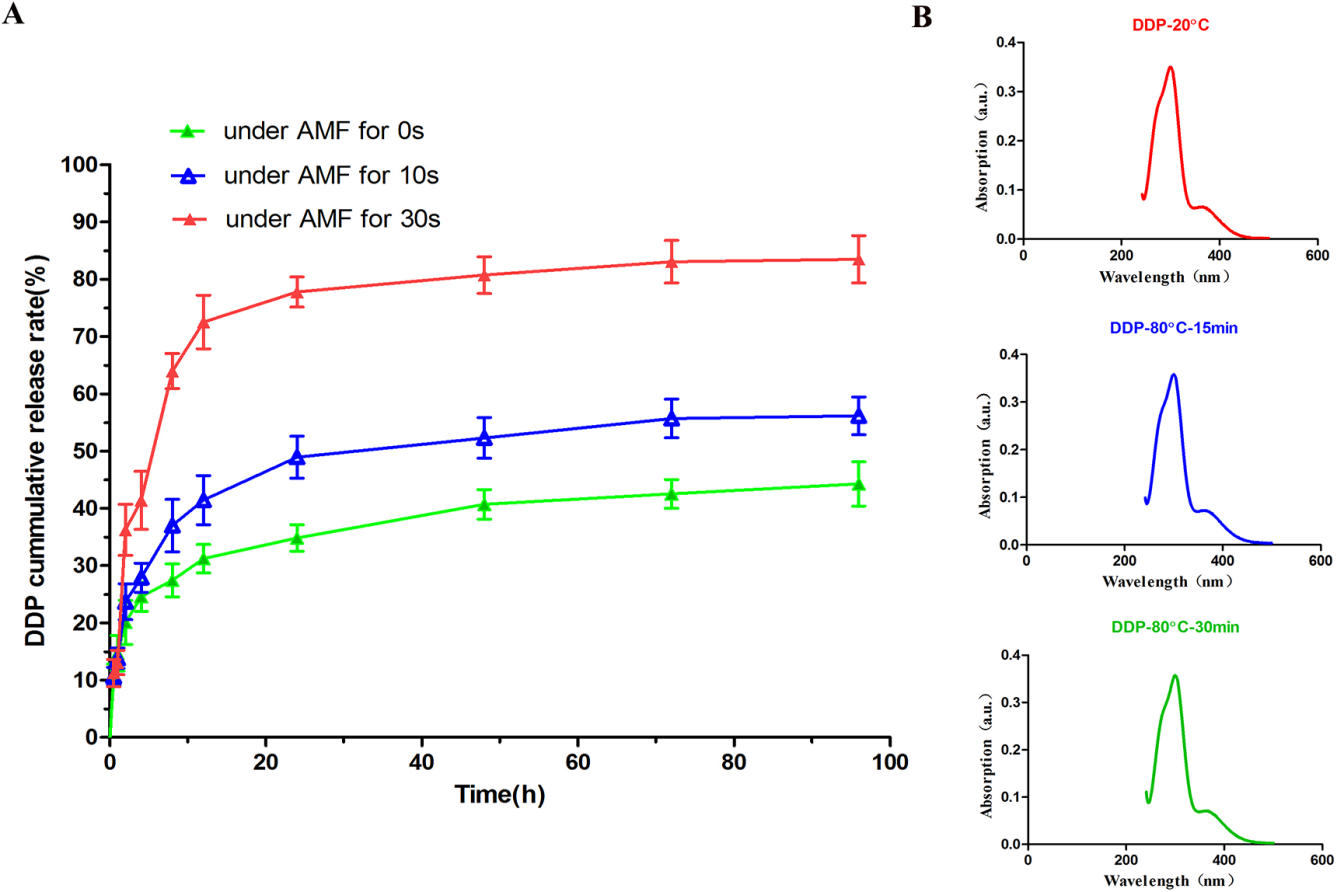
The cumulative release curves and thermal stability of DDP. (A) The DDP cumulative release curves with exposure to AMF for 0 s, 10 s, and 30 s at the time points of 2 h and 8 h, respectively, or without exposure to AMF. (B) UV-vis-NIR absorbance spectrum of DDP aqueous solution (20°C), DDP aqueous solution (80°C, 15 min), and DDP aqueous solution (80°C, 30 min). The maximum absorption peak of DDP aqueous solution remained the same at temperatures of 20°C, 80°C for 15 min, and 80°C for 30 min.

This phase transformation in situ implant can simultaneously act as a medium for both magnetic hyperthermia therapy and local drug carriers. It is highly feasible to fabricate DDP-loaded magnetic implant devices by a solvent method. We investigated the drug release profile of DDP/PLGA-Fe_3_O_4_ when stimulated by AMF. In comparison, a higher drug release was found with exposure to AMF than without exposure to AMF (Fig 5A). In addition, the drug release increased with prolonged time exposure to AMF. The cumulative release of DDP exposed to AMF for 30 s was higher than that exposed to AMF for 10 s at a given point in time (2 h and 8 h), indicating that the magnetic responsive drug release profile is strongly related to the temperature. After 96 h, the cumulative release increased from 44.3 ± 3.9% (not exposed to AMF) to 56.2 ± 3.3% (exposed to AMF for 10 s) and 83.5 ± 8.7% (exposed to AMF for 30 s), respectively, indicating that the release of DDP from DDP/PLGA-Fe_3_O_4_ can be boosted and controlled by the AMF. Hence, we proposed to increase the DDP release using hyperthermia induced by AMF to reduce the dose and minimize the systemic toxicity of DDP. We also validated the thermal stability of DDP at a high temperature by UV-vis spectrophotometry. Fig 5B shows that the maximum absorption peak of DDP aqueous solution at 80°C for 15 min and 30 min were the same as that of DDP aqueous solution at 20°C, which suggests that the drug properties of DDP can be preserved to guarantee its effect during thermal therapy.

### In vitro biocompatibility and cytotoxicity of PLGA-Fe_3_O_4_

Because these magnetic materials are ultimately expected to be injected into patients for tumor therapy, their safety issues are important and should be considerable. We chose biodegradable PLGA and biocompatible NMP to prepare the liquid gel solution. Iron oxide nanoparticles added to the gel exist in nature, have a good biocompatibility profile, and have been approved by the FDA[40]. To further study its application in vivo, we investigated more details about its property. The cytotoxicity of PLGA-30% Fe_3_O_4_ was confirmed using the human ovarian cancer cell line SKOV3 that was incubated with different concentrations of PLGA-30% Fe_3_O_4_ immersed in RPM1640. The results of a CCK8 assay showed that SKVO3 cells maintained a high viability of approximately 89.2% even at a high concentration in immersed solution of 20 μl/ml (S1 Fig). There was no obvious cytotoxicity to SKVO3 cells in various concentrations of immersed solution groups compared to the control group (*P* > 0.05), indicating that the implants were biologically safe.

### US/CT and thermal imaging in vivo

**Fig 6:**
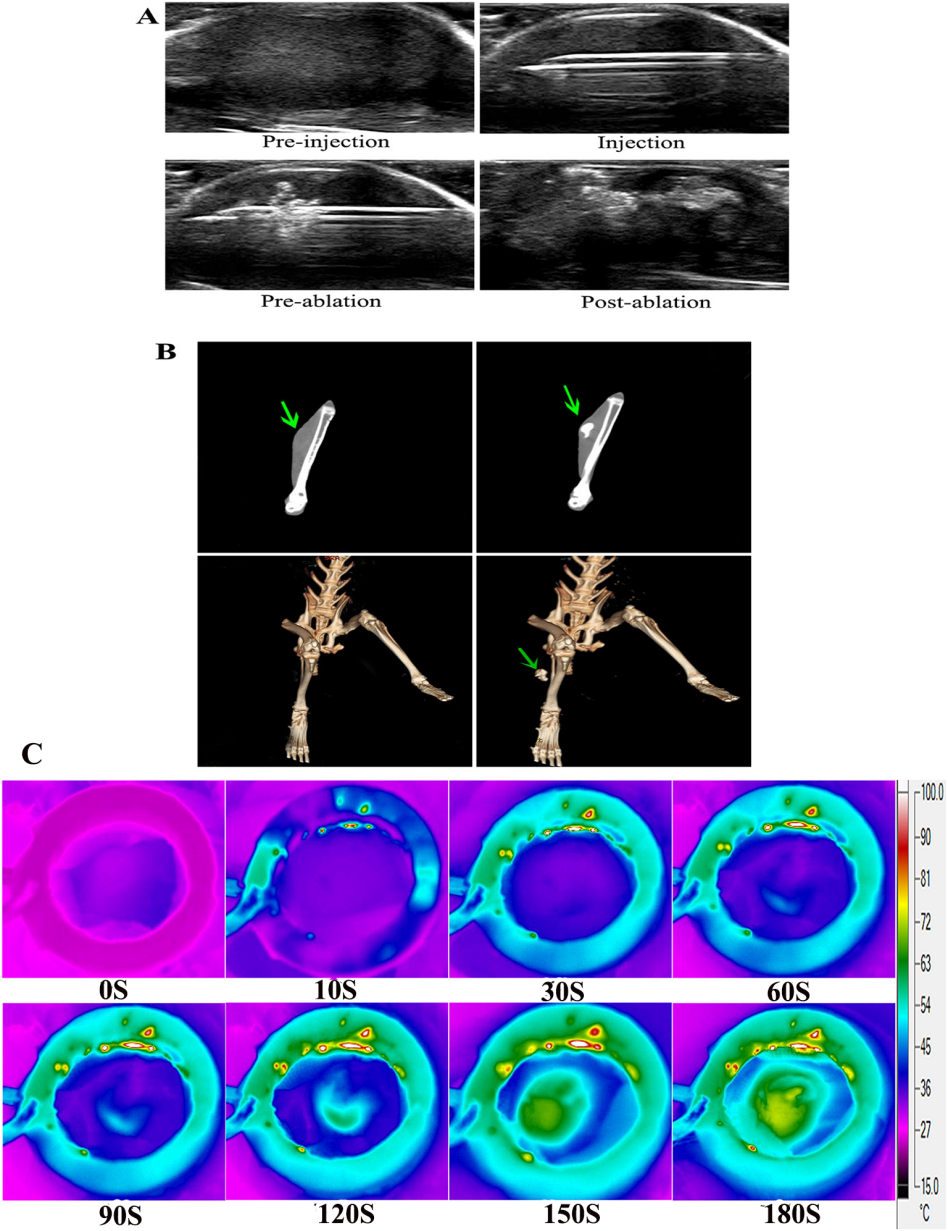
In vivo US, CT and thermal imaging. (A) US images of intratumoral injection procedure and after ablation_._ (B) Plain scanning (the first line) and 3D reconstruction of CT images (the second line) (the green arrows indicate the tumor region before injection and after ablation. (C)Thermal images of the rabbit bearing VX2 transplanted carcinoma in its thigh after the intratumoral injection of 100 μl DDP/PLGA-30% Fe_3_O_4_ and exposed to AMF for 180 s.

The principal problem with thermal is restricting the hyperthermia effect in the region of interest. The accurate guidance is essential for delivering the implants to the target site and monitoring the therapeutic procedure. We explored the imaging ability of DDP/PLGA-Fe_3_O_4_ in vivo. As shown in Fig 6A, the tumor had an elliptical shape and appeared isoechoic in US imaging. DDP/PLGA-Fe_3_O_4_ was visible under US appearing hyperechoic with posterior acoustic shadow. After ablation, the echo intensity in the neighboring tissue increased. We also investigated it by plain CT scanning. In the coronal section images, the tumor was isodense and it was not easy to distinguish it from the surrounding normal tissue. After intratumoral injection, the CT value of the tumor significantly increased like the contrast enhancement effect (Fig 6B). Also, the 3D (3-dimensional reconstruction) images of the CT indicated that the implants were well-distributed in the tumor site without leakage before and after the magnetic heating process, even as the temperature reached approximate 70°C in the tumor, which had a safe record to prevent the adjacent normal tissue from heating, as the thermal images showed in Fig 6C. Hence, DDP/PLGA-Fe_3_O_4_ was visible to US and CT imaging in a liquid or solid state, which showed it had great potential to precisely guide and monitor the therapeutic procedure as a US and CT imaging contrast agent.

### In vivo magnetic-induced chemo-hyperthermia ablation therapy

**Fig 7:**
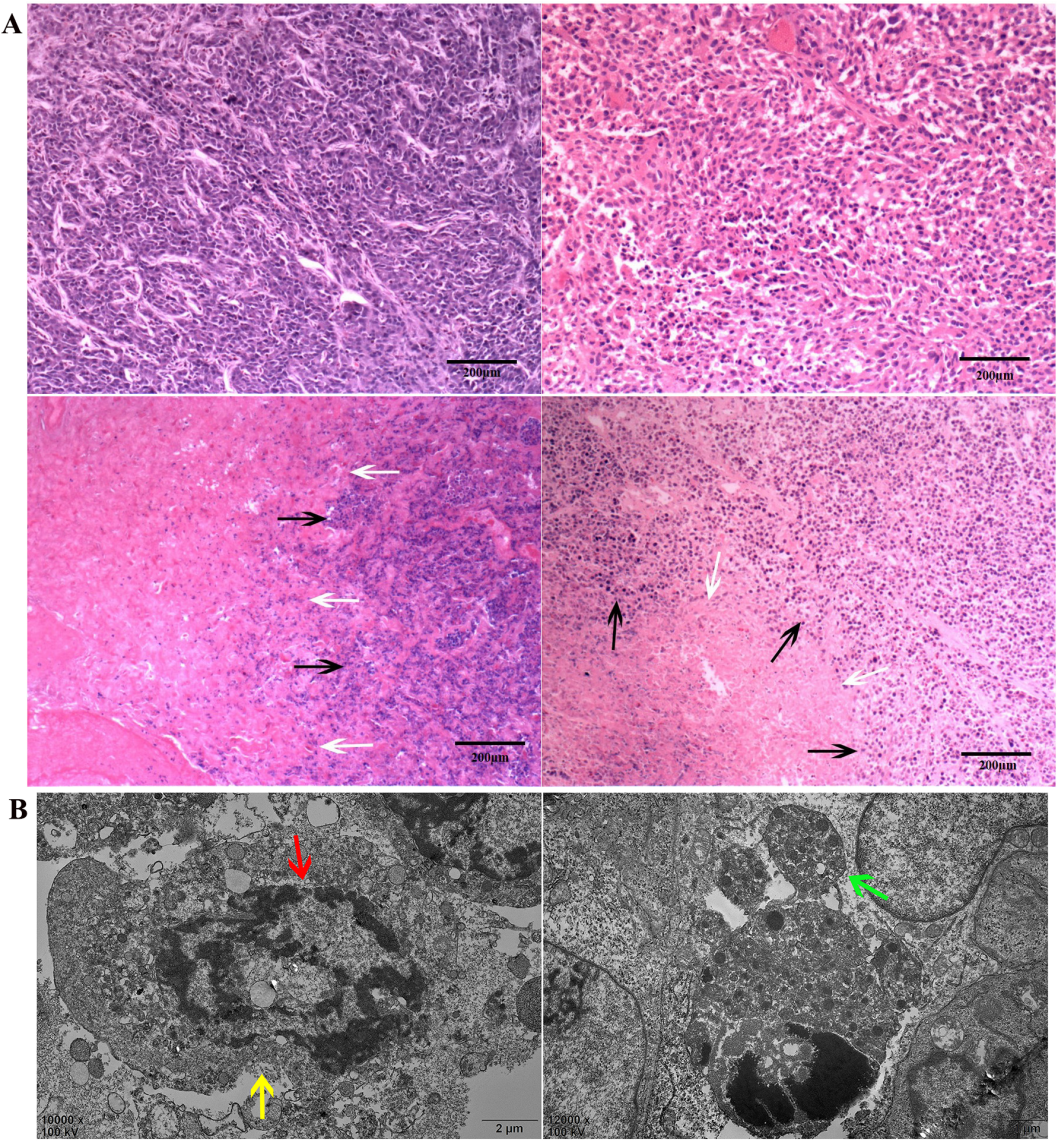
HE staining and TEM images of tumors after therapy. (A) HE staining of tumors in the 4 groups (100 × magnification, scale bar: 200 μm). Black arrows indicate non-ablated tumor tissues, and white arrows indicate necrotic tissues. (B) TEM images of the ablated tumor tissues indicate the ultra-structure changes; green arrows indicate the apoptotic body; the plasma membranes (yellow arrows) and nuclear membranes (red arrows) were interrupted or undefined; and karyolysis and cytoplasm lysis were observed (scale bar: 2 μm).

**Fig 8:**
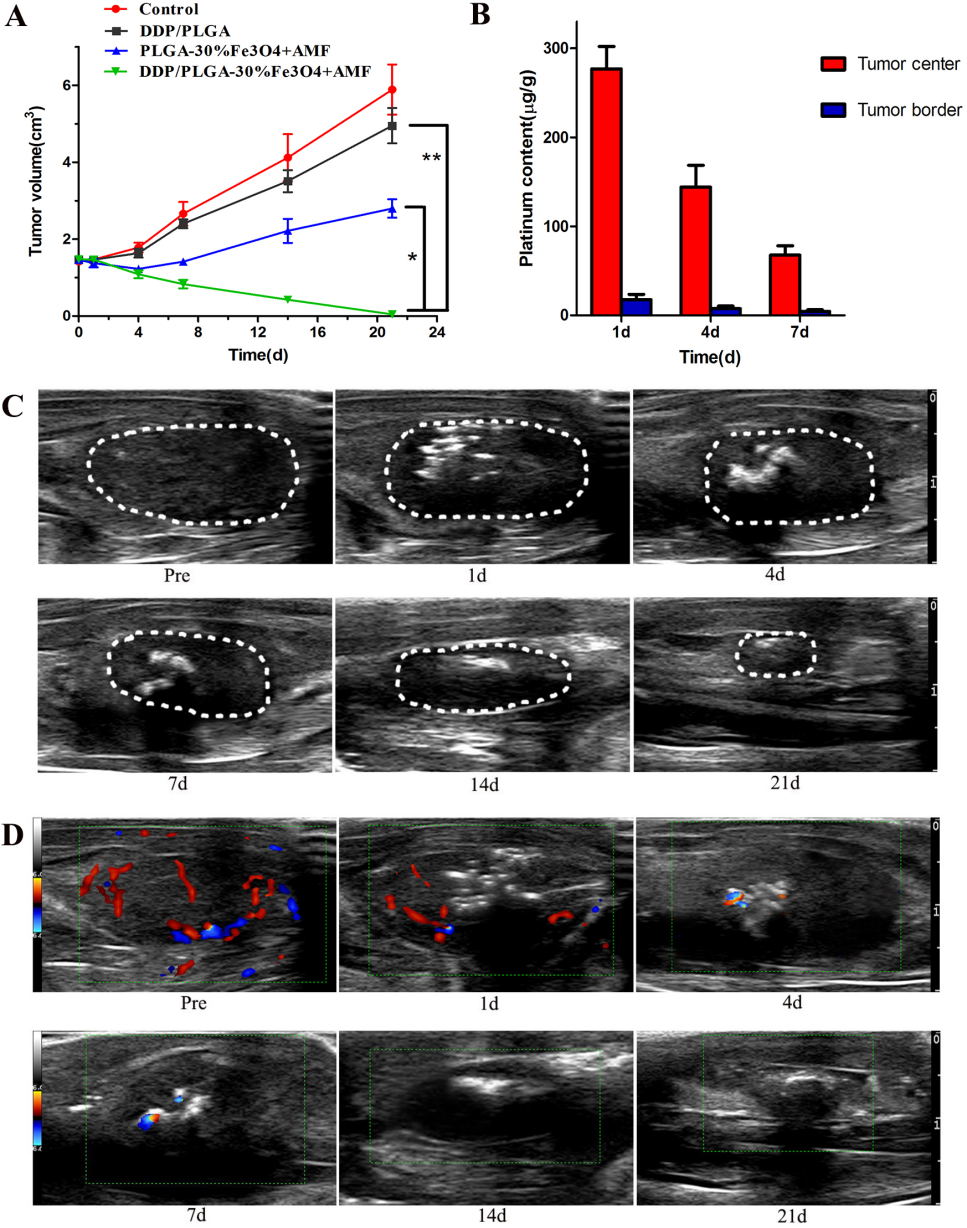
In vivo magnetic thermal therapy and chemotherapy for tumor. (A) The tumor volume–time curve in different groups after ablation (*P < 0.05). (B) Platinum concentration in the center of the tumor and in the edge of the tumor in the DDP/PLGA-30% Fe + AMF group. (C) US 2D images of tumors in the DDP/PLGA-30% Fe + AMF group prior to therapy and on days 1, 4, 7, 14, and 21 after therapy (white circle: the tumor region). (D) CDFI of tumors in the DDP/PLGA-30% Fe + AMF group prior to therapy and on days 1, 4, 7, 14, and 21 after therapy. Behind the hyperechoic implants on the images of days 4 and 7 is the color Doppler twinkling artifact.

Forty-eight rabbits bearing VX2 transplanted carcinoma in their thigh were used to evaluate the anti-cancer effects of DDP/PLGA-30% Fe_3_O_4_. Incomplete ablation often occurs and should be considered, especially when the tumor is large, irregular, and deep inside the tissue. Therefore, we investigated the synergistic efficacy of chemotherapy and magnetic hyperthermia ablation in residual tumors of large animal models. The residual tumor models were built by exposure to AMF for 3 min after injection, which could not produce sufficient heat effects for complete ablation based on the previous experiment. Fig 7A is the HE staining on day 1 after treatment: the edge of the coagulative necrosis in the partially ablated tumor was obvious, which indicated that the residual tumor models were built successfully. Irreversible destruction of cells in the necrotic tissue was observed under TEM, with lysis (red arrow and yellow arrow) and apoptotic body (green arrow) occurrence (Fig 7B). The time-tumor volume curve shows that the tumor volumes in the DDP/PLGA-30% Fe_3_O_4_ + AMF group presented a decreasing trend while the tumor volumes in the 3 other groups continued to increase at varying degrees over the next 21 days after treatment, which indicated that the combination therapy was far more effective than either hyperthermia or chemotherapy alone (Fig 8A). The synergistic anti-cancer effect of DDP is not only determined by the local concentration of drugs, the time of contact with the tumor cell is more important. Sustained drug release formulation may provide better efficacy and fewer side effects. The concentration of platinum in the tumor was detected by ICP-AES. Platinum in the center of the tumor as well as in the edge of the tumor was detected even on day 7 after the treatment. The concentration of platinum decreased over time, demonstrating that DDP was gradually released from the implants (Fig 8B). The sustained release of DDP/PLGA-Fe_3_O_4_ can maintain an adequate DDP concentration in the tumor region over an extended period to eliminate residual tumor cells. Moreover, hyperthermia is able to alter drug distribution and metabolism in the tumor tissue and increase blood vessels and cell membrane permeability, which is feasible for the penetration of chemotherapeutic drugs into tumor cells and maintains a high intracellular drug concentration. In addition, hyperthermia is able to reduce resistance to chemotherapeutic drugs because of the damage to multi-drug resistance proteins on the cell membrane, and decreased DNA-repair enzyme activity induced by hyperthermia results in inhibition of repair of DNA single and double strands. All of these biomedical and molecular biological data explain the enhanced synergistic effect of DDP.

US 2D images and color Doppler flow imaging (CDFI) in the DDP/PLGA-30% Fe_3_O_4_ + AMF group showed that the tumor dimension apparently shrank; the blood flow in the tumor reduced significantly compared to pre-treatment and no blood flow signal in and around the tumor tissue had been detected since 4 days after treatment (Fig 8C and 8D). Moreover, the hyperechoic signals derived from the implants in the tumor decreased over time. It is observed that the hyperechoic region in the tumor shrank. Only few hyperechoic signals were observed in the grayscale images on day 21 after treatment, indicating that the material can gradually biodegrade with time in vivo, which can avoid the adverse effects caused by the foreign matter being in the body for a long time. Due to their biodegradable and injectable nature, the implants do not require surgery for insertion or removal from the body, which improves the patients’ comfort and compliance.

**Fig 9:**
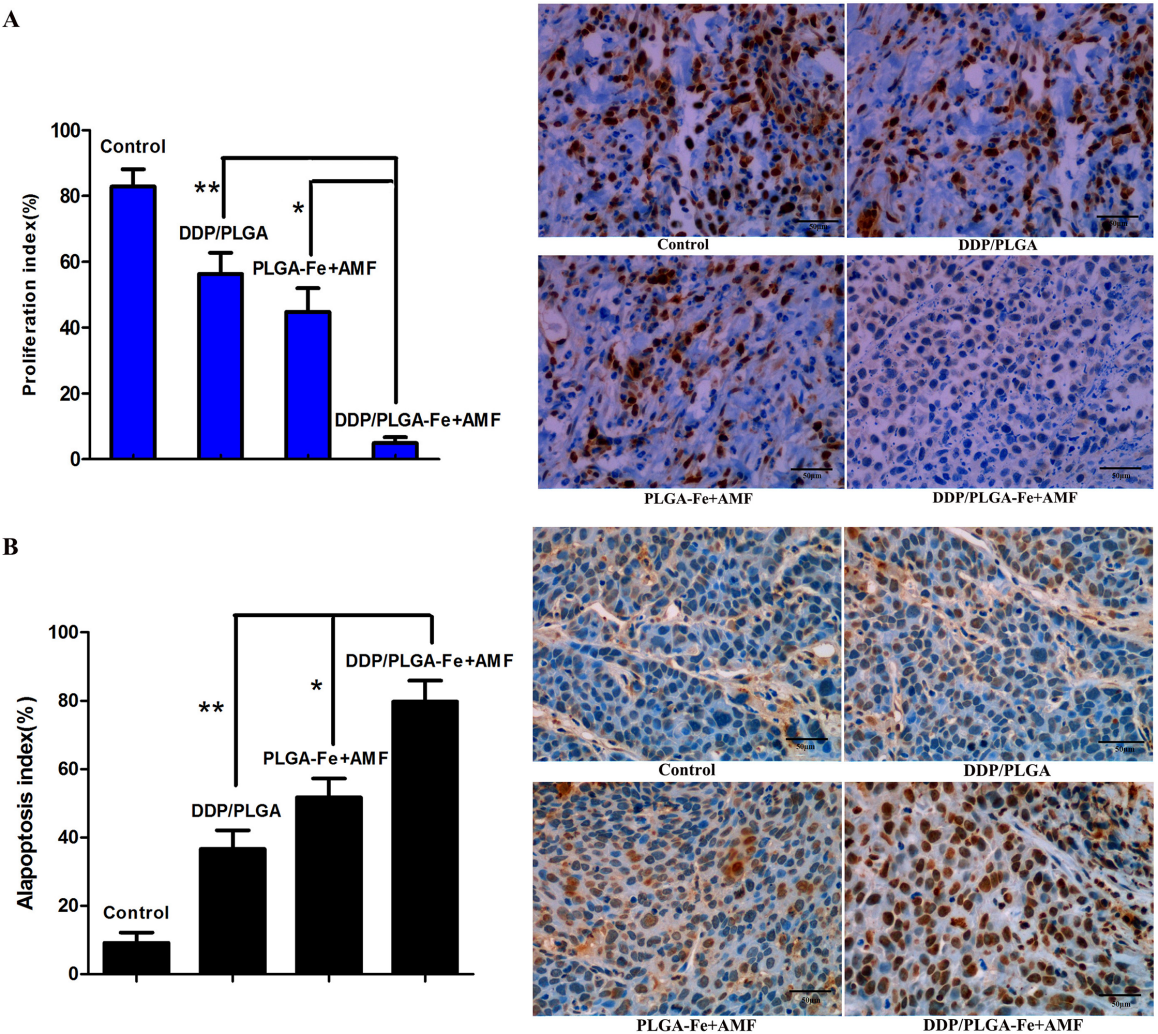
Immunohistochemical staining of tumors after therapy in each group. (A) PCNA staining and the PI of tumor tissues in each group, 400 ×magnification, scale bar: 50 μm, *P < 0.05, and **P < 0.05. The asterisk indicates the significant difference in the PI in each group. The nucleus that appears brown is a PCNA-positive cell, while blue is negative. (B) TUNEL staining and the AI of tumor tissues in each group, 400 × magnification, scale bar: 50 μm, *P < 0.05, and **P < 0.05. The asterisk indicates the significant difference in the AI in each group. The nucleus that appears brown is a TUNEL-positive cell, while blue is negative.

Additionally, the anti-tumor effect was assessed by immunohistochemical staining. Fig 9A shows that the PI of PCNA in the DDP/PLGA-30% Fe_3_O_4_+AMF group was the lowest in comparison with that of the other groups (*P* < 0.05). Fig 9B shows that the AI of TUNEL in the DDP/PLGA-30% Fe_3_O_4_ + AMF group was the highest compared to that of the other 3 groups (*P* < 0.05). However, there was no significant difference between the PLGA/NMP and DDP/PLGA groups, indicating that the tumors were not apparently affected by the solo chemotherapy at this low dose. These results demonstrated that the therapeutic efficacy of the DDP/PLGA-30% Fe_3_O_4_ + AMF group was superior to the other groups and it can be developed as a highly promising agent to enhance the effects of this therapy on tumors.

**Fig 10:**
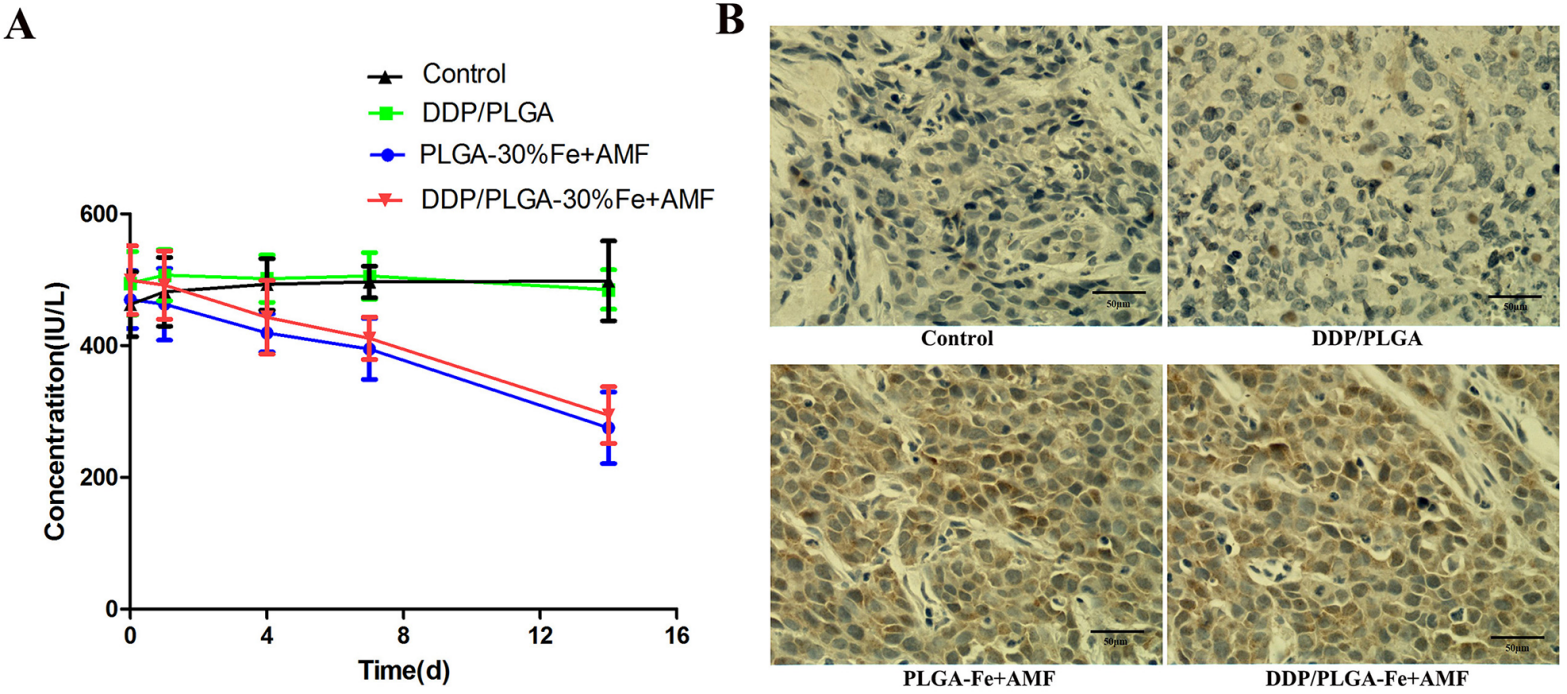
Hyperthermia can stimulate the host immune response. (A) The IL-2R level of rabbit serum in different groups before and after treatment. (B) The expression of HSP70 in different groups detected by immunohistochemistry staining of tumor tissues on day 1 after treatment. The nucleus that appears brown is an HSP70-positive cell, while the blue is negative (scale bar: 50 μm).

It is reported that the synergistic effects of hyperthermia/chemotherapy are due to hyperthermia-induced apoptosis, the immune response, and increased blood flow. Hyperthermia can lead to direct damage of tumor cells and meanwhile motivate the immune system to induce distant lesions, which is known as the abscopal effect in cancer treatment. IL-2R is known to be an important cytokine with many biological functions and specific binding to interleukin-2 (IL-2). IL-2R like blocking factor can reduce the immune response depending on IL-2 and inhibit the immune reaction of activated lymphocytes. Fig 10A shows that the IL-2R level of the serum in the DDP/PLGA-30% Fe_3_O_4_ + AMF group and the PLGA-30% Fe_3_O_4_ + AMF group decreased with time and were eventually lower than those in the groups without exposure to AMF. However, no significant changes of the IL-2R level were observed in the groups without exposure to AMF during the treatment, indicating that hyperthermia could improve the host immune state and enhance anti-tumor treatment. An interesting aspect to take into consideration during hyperthermia is the expression of (HSPs). It is reported that hyperthermia-induced changes in immune function are directly related to the emergence of HSPs, which are released from stressed or dying cells. Released HSPs activate natural killer cells and dendritic cells to secrete cytokines and transform them into antigen-presenting cells[41]. Ito et al. injected recombinant mouse HSP70 protein into melanoma nodules in C57BL/6 mice in situ and implemented magnetic fluid hyperthermia. The combined treatment strongly inhibited tumor growth over a 30-day period and complete regression of tumors was observed in 20% of mice. It was also found that systemic antitumor immunity was induced in the cured mice, which demonstrated the antitumor immunity induced by hyperthermia was enhanced[42]. On the other hand, it is known that thermoresistance occurs as a response to thermal stress and arises from the synthesis of HSPs to protect cells from apoptosis may by preventing the unfolding and aggregation of key proteins when they are exposed to a mild temperature [43]. HSP70 is considered to be the most important stress protein in the heat-shock protein family, which closely links to stress-mediated chaperonin functional responses, but the exact mechanisms is unclear. We investigated the expression of HSP70 by immunohistochemistry staining of the tumor tissues on the first day. The temperature of the tumor region increased rapidly and reached in excess of at least 60°C for a few minutes, which was a lethal thermal stress that caused the tumor cells coagulative necrosis, consequently, HSPs cannot be expressed. We predicted that the increasing expression of HSP70 may stem from the surrounding non-complete ablated tumor tissue. Fig 10B shows that the expression intensity and expression rate in 2 hyperthermia groups were much higher than that in the control group and in the chemotherapy group. The expression of HSP70 was consistent with the IL-2R level in each group, indicating the increasing expression of HSP70 after ablation can stimulate the host immune system. Thus, this additional feature opens up possibilities for the development of multi-functional and multi-therapeutic approaches for treating tumors.

## Conclusions

In conclusion, we have successfully designed a smart injectable magnetic hyperthermal ablation material PLGA@Fe_3_O_4_ loading cisplatin chemotherapeutic drug, which shows excellent heating ability for rabbit VX2 tumor thermal ablation and controlled drug release performance when exposed to AMF. Finally, the therapeutic efficiency was improved by the synergy of DDP and hyperthermia as well as improved immune response. The whole process of therapy can be monitored by US and CT imaging. The new and powerful phase transformation in situ implants is a promising minimally invasive agent in the treatment of tumors.

## Supporting information

S1 Fig The viability of SKVO3 cells. The viability of cells in different concentrations of an immersed solution of DDP/PLGA-30% Fe_3_O_4_ (5 μl/ml, 10 μl/ml, and 20 μl/ml, respectively).
(TIF)

S2 Fig The corresponding time-temperature curve of the rabbit VX2 tumor. After the intratumoral injection of 100 μl DDP/PLGA-30% Fe_3_O_4_ and exposed to AMF for 180 s, the temperature of the tumor reached to 72.3 ± 2.2°C in 180 s, which was higher than the temperature for tumor coagulative necrosis.
(TIF)

S1 Table Endpoints of animals during the experiment. (DOCX)
