# Supplementary figures and images for: Injectable PLGA/Fe3O4 implants carrying cisplatin for synergistic magnetic hyperthermal ablation of rabbit VX2 tumor
Source: PLoS One. 2017 May 4;12(5):e0177049. doi: 10.1371/journal.pone.0177049 (PMC5417648; doi:10.1371/journal.pone.0177049)

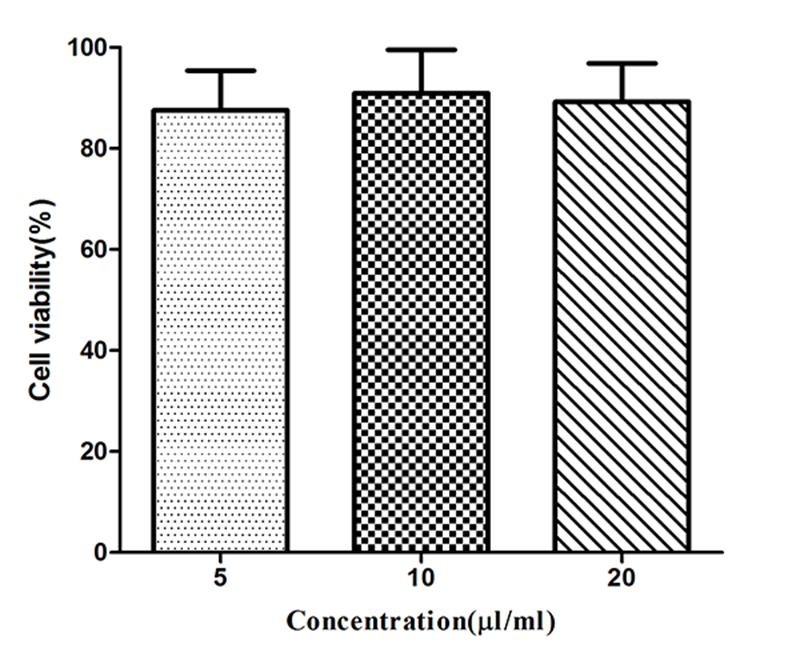

Supplement: S1 Fig — The viability of cells in different concentrations of an immersed solution of DDP/PLGA-30% Fe3O4 (5 μl/ml, 10 μl/ml, and 20 μl/ml, respectively). (TIF) [file pone.0177049.s001.tif]

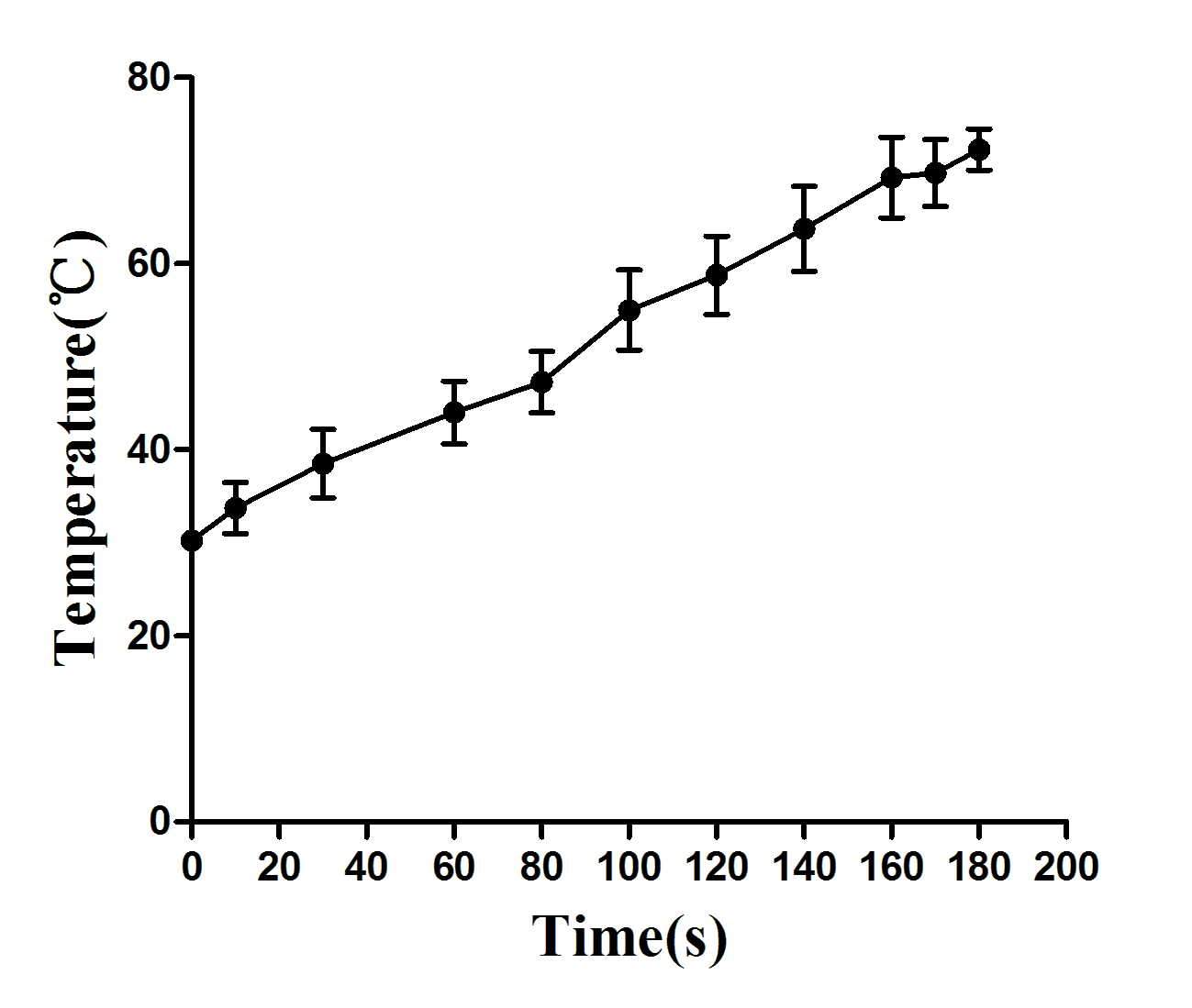

Supplement: S2 Fig — After the intratumoral injection of 100 μl DDP/PLGA-30% Fe3O4 and exposed to AMF for 180 s, the temperature of the tumor reached to 72.3 ± 2.2°C in 180 s, which was higher than the temperature for tumor coagulative necrosis. (TIF) [file pone.0177049.s002.tif]
