# Supplementary material for: Injectable PLGA/Fe3O4 implants carrying cisplatin for synergistic magnetic hyperthermal ablation of rabbit VX2 tumor
Source: PLoS One. 2017 May 4;12(5):e0177049. doi: 10.1371/journal.pone.0177049 (PMC5417648; doi:10.1371/journal.pone.0177049)
Supplement: S1 Table — (DOCX) [file pone.0177049.s003.docx]

**S1 Table** **Endpoints of animals during the experiment.**

| Group | Sacrifice Day 1 | Sacrifice Day 4 | Sacrifice Day 7 | Ethical Sacrifice Day 21 |
| --- | --- | --- | --- | --- |
| PLGA/NMP | 3 | 3 | 3 | 3 |
| DDP/PLGA | 3 | 3 | 3 | 3 |
| PLGA-30% Fe_3_O_4_ + AMF | 3 | 3 | 3 | 3 |
| DDP/PLGA-30% Fe_3_O_4_ + AMF | 3 | 3 | 3 | 3 |
| Total | 12 | 12 | 12 | 12 |
